# Species-Specific Shifts in Diurnal Sap Velocity Dynamics and Hysteretic Behavior of Ecophysiological Variables During the 2015–2016 El Niño Event in the Amazon Forest

**DOI:** 10.3389/fpls.2019.00830

**Published:** 2019-06-28

**Authors:** Bruno O. Gimenez, Kolby J. Jardine, Niro Higuchi, Robinson I. Negrón-Juárez, Israel de Jesus Sampaio-Filho, Leticia O. Cobello, Clarissa G. Fontes, Todd E. Dawson, Charuleka Varadharajan, Danielle S. Christianson, Gustavo C. Spanner, Alessandro C. Araújo, Jeffrey M. Warren, Brent D. Newman, Jennifer A. Holm, Charles D. Koven, Nate G. McDowell, Jeffrey Q. Chambers

**Affiliations:** ^1^National Institute of Amazonian Research (INPA), Manaus, Brazil; ^2^Climate and Ecosystem Sciences Division, Lawrence Berkeley National Laboratory, Berkeley, CA, United States; ^3^Department of Integrative Biology, University of California, Berkeley, Berkeley, CA, United States; ^4^Embrapa Amazônia Oriental, Belém, Brazil; ^5^Environmental Sciences Division and Climate Change Science Institute, Oak Ridge National Laboratory, Oak Ridge, TN, United States; ^6^Earth and Environmental Sciences Division, Los Alamos National Laboratory, Los Alamos, NM, United States; ^7^Pacific Northwest National Laboratory, Richland, WA, United States; ^8^Department of Geography, University of California, Berkeley, Berkeley, CA, United States

**Keywords:** tropical forests, sap velocity, stomatal conductance, direct solar radiation, vapor pressure deficit, leaf temperature, hysteresis

## Abstract

Current climate change scenarios indicate warmer temperatures and the potential for more extreme droughts in the tropics, such that a mechanistic understanding of the water cycle from individual trees to landscapes is needed to adequately predict future changes in forest structure and function. In this study, we contrasted physiological responses of tropical trees during a normal dry season with the extreme dry season due to the 2015–2016 El Niño-Southern Oscillation (ENSO) event. We quantified high resolution temporal dynamics of sap velocity (V_s_), stomatal conductance (g_s_) and leaf water potential (Ψ_L_) of multiple canopy trees, and their correlations with leaf temperature (T_leaf_) and environmental conditions [direct solar radiation, air temperature (T_air_) and vapor pressure deficit (VPD)]. The experiment leveraged canopy access towers to measure adjacent trees at the ZF2 and Tapajós tropical forest research (near the cities of Manaus and Santarém). The temporal difference between the peak of g_s_ (late morning) and the peak of VPD (early afternoon) is one of the major regulators of sap velocity hysteresis patterns. Sap velocity displayed species-specific diurnal hysteresis patterns reflected by changes in T_leaf_. In the morning, T_leaf_ and sap velocity displayed a sigmoidal relationship. In the afternoon, stomatal conductance declined as T_leaf_ approached a daily peak, allowing Ψ_L_ to begin recovery, while sap velocity declined with an exponential relationship with T_leaf_. In Manaus, hysteresis indices of the variables T_leaf_-T_air_ and Ψ_L_-T_leaf_ were calculated for different species and a significant difference (*p* < 0.01, α = 0.05) was observed when the 2015 dry season (ENSO period) was compared with the 2017 dry season (“control scenario”). In some days during the 2015 ENSO event, T_leaf_ approached 40°C for all studied species and the differences between T_leaf_ and T_air_ reached as high at 8°C (average difference: 1.65 ± 1.07°C). Generally, T_leaf_ was higher than T_air_ during the middle morning to early afternoon, and lower than T_air_ during the early morning, late afternoon and night. Our results support the hypothesis that partial stomatal closure allows for a recovery in Ψ_L_ during the afternoon period giving an observed counterclockwise hysteresis pattern between Ψ_L_ and T_leaf_.

## Introduction

Evapotranspiration by terrestrial ecosystems delivers an estimated 62,000 km^3^ of water to the atmosphere every year, with the majority associated with plant transpiration (Jasechko et al., 2013). In the Amazon Basin, an estimated 25–50% of precipitation is recycled back to the atmosphere through forest transpiration (Eltahir and Bras, 1994; Chambers and Artaxo, 2017), with important implications for the interactions between the biosphere and atmosphere (Araújo et al., 2002; Negrón-Juárez et al., 2007). Under climate change scenarios, vegetation resilience will depend on the capacity to exploit water resources (Grossiord et al., 2017), and a mechanistic understanding of the water cycles from individual trees to landscape scales is necessary in order to predict changes in the forest structure (Chambers et al., 2014).

At the leaf level, transpiration flux is a function of vapor pressure deficit (VPD) between the leaf and the air and stomatal conductance (g_s_), according to Fick’s law of diffusion (Costa et al., 2010). Although numerous environmental factors influence g_s_, net radiation, VPD and soil moisture are often considered the most important (Jarvis, 1976; Jones, 1998; Lloyd and Farquhar, 2008; Daloso et al., 2017). High leaf temperatures (T_leaf_) and VPD are known to induce stomatal closure in order to minimize excessive water loss (Farquhar, 1978; Meinzer et al., 1993; Tinoco-Ojanguren and Pearcy, 1993; Oren et al., 1999b; McAdam et al., 2016; Brodribb et al., 2017). The degree of stomatal closure is a balancing act between preventing hydraulic damage while still allowing enough CO_2_ influx for carbon fixation to avoid carbon-starvation (Adams et al., 2017). In addition, stomatal closure limits water lost through transpiration, and thereby indirectly regulates leaf temperatures. Given that the tropics have among the narrowest seasonal temperature range of any biome globally, they may be particularly sensitive to even small increases in temperature associated with climate change (Field et al., 2014). Indeed, rising temperature and VPD are environmental factors clearly associated with increased tree mortality in the tropics (McDowell et al., 2018), with more pronounced impacts during extreme drought events in the Amazon forest, such as the El Niño-Southern Oscillation (ENSO). This reinforces the importance of having more studies that investigate the effect of these variables (temperature and VPD) in the tropics, especially focusing on comparisons between two distinct periods, such as normal years (control scenario) and years with ENSO. This kind of approach can be considered as a “natural experiment” and allow to expand our understanding of the coupling of tree water use (and concurrent carbon uptake) and the environmental factors that affect stomatal conductance – solar radiation, CO_2_, air temperature, leaf temperature and humidity.

The water potential gradient that regulates water movement through trees is anchored by soil moisture availability on one end, and atmospheric moisture availability VPD on the other. VPD is indirectly estimated from measurements of relative humidity (R_H_) and air temperature (T_air_) using micrometeorological sensors (Ewers and Oren, 2000). However, as T_leaf_ and T_air_ can differ by several degrees, the use of T_leaf_ instead of T_air_ to calculate VPD (ΔVPD) results in a more accurate representation of the true water vapor pressure gradient between the substomatal cavity and the boundary layer of the air near the leaf surface (Ewers and Oren, 2000). Therefore, T_leaf_ measurements are vital for better interpretation of plant hydraulic responses to environmental drivers in order to develop more accurate earth system models (ESMs) (Michaletz et al., 2016). However, sap velocity, T_leaf_ and environmental drivers are rarely measured together, especially in the tropics where the canopy layers are often hard to access (Chave et al., 2005; Segura and Kanninen, 2005). Thus, the response of a plant’s transpiration to changes in environmental and physiological conditions remains highly uncertain in ESMs (Jasechko et al., 2013).

In relation to environmental drivers, clockwise hysteresis patterns between sap flow and VPD have been reported with higher sap flow rates during the morning period relative to the afternoon (O’Brien et al., 2004; Zeppel et al., 2004; Zhang et al., 2014). In addition, a counterclockwise hysteresis pattern has been observed in tropical and temperate forests when sap flow is plotted as function of irradiance (O’Brien et al., 2004; Zeppel et al., 2004; Bretfeld et al., 2018; Brum et al., 2018). In the case of transpiration, it has been established that the hysteresis phenomena are influenced by the temporal lag between solar radiation, which tends to peak in the late morning to mid-day, and VPD which tends to peak in the early afternoon (O’Brien et al., 2004; Zeppel et al., 2004; Zhang et al., 2014; Novick et al., 2016). Also, hysteresis between sap flux and environmental drivers are influenced by the stored stem water and the time lag between basal sap velocity and upper canopy transpiration, as an effect of hydraulic capacitance and resistance (Phillips et al., 1997; Ward et al., 2012). However, coupled field observations of physiological and environmental variables that include not only diurnal sap velocity and environmental driving data, but also concurrent leaf level data such as g_s_, T_leaf_ and Ψ_L_ has been very limited in the tropics. Yet such data, are needed to verify the relationships between V_s_ and environmental/physiological drivers in tropical forests.

In this study, we present *in situ* field observations of environmental (direct solar radiation, T_air_ and VPD) and physiological (V_s_, g_s_, and Ψ_L_) variables and their correlations with T_leaf_ during the 2015–2016 ENSO. In order to observe the interactions between physiological variables and fast changing environmental conditions, we collected high temporal frequency data (15–60 min) in two primary rainforest sites located in the Eastern (Santarém) and in the Central (Manaus) Amazon. Since the 2015–2016 ENSO event was the warmest period in the Amazon forest over the past 13 years (Fontes et al., 2018), we expected peak T_leaf_ to increase and subsequently hysteretic behavior of water use vs. T_leaf_ to become more pronounced. In this study, we explored the mechanisms that regulate tree transpiration and the diurnal hysteresis patterns between physiological and environmental variables to contrast different tree species responses to the extreme 2015 dry season (ENSO) and a normal 2017 dry season (“control scenario”).

## Materials and Methods

### Study Area

The field activities occurred in two sites near the cities of Manaus and Santarém, Brazil (Supplementary Figure S1). Near the city of Manaus, trees with leaves accessible from the K-34 walkup tower were selected for study. The 50-meter tall K-34 tower is located at the Reserva Biológica do Cuieiras, also known as ZF-2, and contains roughly 22,000 ha adjacent to extensive areas of undisturbed tropical forest (Araújo et al., 2002). The mean value of rainfall is ∼2,500 mm year^-1^ with the driest months of the year concentrated from July to September (Araújo et al., 2002). Field data were collected at the K-34 tower site between July 01, 2015 and December 01, 2017.

Near the city of Santarém, four trees near the K-67 triangle tower were selected for study, located in the Tapajós National Forest with approximately 527,000 ha near the Santarém-Cuiabá highway (BR-163). The K-67 tower is located ∼6 km west of the BR-163 and ∼6 km east of Tapajós river, in an area of largely contiguous forest from north to south (Hutyra et al., 2007). The site receives ∼2,000 mm year^-1^ of rainfall and has a five-month dry season from mid-July to mid-December (Saleska et al., 2003; Wu et al., 2017). In Santarém, field data were collected during the period of April 01, 2016 to December 31, 2016.

### Species Selection

Different species were selected in a plateau area of Tapajós National Forest (Santarém) and Reserva Biológica do Cueiras (ZF-2 – Manaus) (eight species in total; Table 1). Tree selection criteria were based on the proximity of the crowns to the two canopy access towers (K-34 and K-67). This approach enabled measurement of physiological variables including sap velocity at breast height, T_leaf_, g_s_, and Ψ_L_ from leaves at the top of the crowns, together with environmental variables including direct solar radiation, T_air_, and R_H_ above the canopy (Supplementary Figure S2).

**Table 1:** List of tree species instrumented with sap velocity and T_leaf_ sensors in Manaus and Santarém.

Site	Tree species	Family	DBH (cm)	Height (m)	IRR viewing height (m)	IRR viewing angle	T_leaf_ target area (m^2^)
Manaus K-34	*^∗^Eschweilera cyathiformis* S.A.Mori	Lecythidaceae	14.3	19.8	3.0	25°	2.41
Manaus K-34	*^∗∗^Pouteria anomala* (Pires) T.D.Penn.	Sapotaceae	35.3	31.0	0.2	10°	0.02
Manaus K-34	*^∗∗^Pouteria erythrochrysa* T.D.Penn.	Sapotaceae	36.5	29.3	0.2	10°	0.02
Manaus K-34	*^∗∗^Couepia longipendula* Pilg.	Chrysobalanaceae	28.1	23.9	0.6	10°	0.19
Santarém K-67	*Erisma uncinatum* Warm.	Vochysiaceae	94.8	39.2	6.5	54°	49.03
Santarém K-67	*Lecythis* sp. Loefl.	Lecythidaceae	81.1	36.4	6.7	63°	18.13
Santarém K-67	*Chamaecrista xinguensis* (Ducke) H.S.Irwin & Barneby	Fabaceae	75.3	29.5	6.2	58°	65.44
Santarém K-67	*^∗^Manilkara* sp. Adans.	Sapotaceae	40.0	30.0	–	–	–


*Also, are shown the tree family, DBH, tree height, IRR viewing height, IRR viewing angle, and T_*leaf*_ target area. ^∗^Species with diurnal measurements of g_*s*_. ^∗∗^Species with diurnal measurements of Ψ_*L*_.*

### Sap Velocity (V_s_) Measurements

One heat pulse sap velocity sensor (SFM1, ICT international^®^) was installed per tree near breast height (DBH) following the protocols previously described by Christianson et al. (2017). The SFM1 sensor consists of a heater and two temperature-sensing probes to determine sap velocity (cm h^-1^) at 0.75 cm depth in the stem using the heat ratio method (Burgess et al., 2001; Green et al., 2003; Steppe et al., 2010). The heater needle was configured to emit a 20 Joule pulse of thermal energy every 15 min (sap heat ratio measurements for 5 min 32 s following the pulse). Biophysical characteristics (diameter and bark thickness) for each tree were used as input into the Sap Flow Tool version 1.4.1 (Phyto-IT^®^) to calculate sap velocity from raw data downloaded from the SFM1 sensors in the field.

The heat pulse method can be used for accurate measurements of sap flow (Lambers et al., 2008), but this method is unable to measure low rates due to its inability to distinguish heat-pulse velocities below a threshold velocity of 3–4 cm h^-1^ (Green et al., 2003). The probe spacing is also an important parameter and the sap velocity (V_s_) is dependent upon the exact distance between needles as the following equation shows:

(1) $$V_{S}=\frac{k}{x}\ln\left(\frac{v_{1}}{v_{2}}\right)\;*\;3600\;\mathrm{cm}\;{\mathrm{hr}}^{-1}$$

where: *k* is the thermal diffusivity of wet wood; *x* is the distance between the heat source (heater) and temperature sensors; *v_1_* and *v_2_* are the increases in temperature (from ambient) at equidistant points downstream and upstream from the heater.

In this study, we used the factory default setting of 5 mm of needle spacings, as recommended by the manufacturer (Burgess and Downey, 2014), using a metal drill guide to ensure equidistant sensor placing. This 5 mm spacing is suitable to a theoretical maximum of 54 cm h^-1^ (Burgess and Downey, 2014).

It should be noted that following the protocols of 5 mm probe spacing of the SFM1 sensors, it is possible that the instrumental maximum was reached at relatively modest flows (16 cm h^-1^ for *Pouteria anomala* for example) on some days. Other available methods to estimate sap flow like the thermal dissipation method and the heat field deformation also underestimate V_s_, where the error tends to increase with further increases in V_s_ (Steppe et al., 2010). However, evidence that the maximum observed V_s_ was not due to a sensor saturation includes: (1) different maximum values of V_s_ between species (plateau in the scatter plots); (2) the state theoretical maximum of 54 cm h^-1^ of the ICT user manual; and (3) on the same trees (Grossiord et al., under review) found no statistical difference between sap velocities determined by ICT and Granier sensors.

### T_leaf_, T_air_, VPD and Direct Solar Radiation Measurements

To measure T_leaf_, a single infrared radiometer sensor (IRR SI-111 analogic for the species *P. anomala, Pouteria erythrochrysa*, and *Couepia longipendula* or IRR SI-131 digital for the species *Eschweilera cyathiformis, Erisma uncinatum, Lecythis* sp. and *Chamaecrista xinguensis*, Apogee^®^) was positioned from the tower’s structure with the field of view targeting the top of individual tree crowns (one IRR sensor per tree). Five-min averages of T_leaf_ were recorded using a CR-3000 (Campbell Scientific^®^ for the SI-111 sensors) and EM-50 (Decagon^®^ for the SI-131 sensors) dataloggers. The sensors were positioned with the viewing heights and viewing angles listed in Table 1. The field of view of each sensor (T_leaf_ target area) was calculated using the IRR calculator available in the website^1^. To validate the infrared radiometer sensors installed on the two sites, T_leaf_ measurements were made using Teflon insulated type T thermocouples (OM-CP-OCTTEMP-A Nomad^®^, Omega Engineering) directly attached to the abaxial side of the leaf using a breathable white tape and configured to register measurements every 15 s (Supplementary Figure S3). In addition, in Manaus direct solar radiation (W m^-2^) with 5-min averages were collected at 35.0 m above the canopy using a SPN1-Sunshine Pyranometer (Delta-T Devices^®^). T_air_ and R_H_ data were obtained using a thermohygrometer (HC2S3, Campbell Scientific^®^) installed above the canopy at 51.1 m height on the K-34 tower structure.

In this study, a more accurate physiological approach to estimate VPD was applied. The Tetens equation was used to calculate the saturation vapor pressure of the air (e_o_) using the variables air temperature (T_air_) and relative humidity of the air (RH_o_) (Eq. 2). To estimate the saturation vapor pressure inside the substomatal chamber (e_i_) the Tetens equation was also used replacing T_air_ by T_leaf_ (Eq. 3). The relative humidity inside the substomatal cavity (RH_i_) was assumed to be equal to 1 as demonstrated by many studies (Ward and Bunce, 1986; Buckley et al., 2017; Cernusak et al., 2018). With these variables it was possible to estimate the VPD difference between the substomatal chamber (e_i_ × RH_i_) and the atmosphere (e_o_ × RH_o_) (Eq. 4).

(2) $$e_{o}=0.611\;\times \;10^{\left(\frac{7.5\;x\;Tair}{Tair+237.3}\right)}$$

(3) $$e_{i}=0.611\;\times \;10^{\left(\frac{7.5\;x\;Tleaf}{Tleaf+237.3}\right)}$$

(4) $$\Delta VPD=(e_{i}\;\times \;RH_{i})-(e_{o}\;\times \;RH_{o})$$

where: ΔVPD is the leaf-to-air water VPD (kPa); e_o_ is the air saturated water vapor pressure (kPa); e_i_ is the saturated water vapor pressure inside the substomatal chamber (kPa); RH_i_ is the relative humidity inside the substomatal cavity which is assumed to be equal to 1. RH_o_ is the relative humidity of the air near the leaf surface (expressed as a decimal); T_leaf_ is the leaf temperature in °C and T_air_ is the air temperature in °C.

### Stomatal Conductance (g_s_) Measurements

Diurnal observations of g_s_ were made on upper canopy leaves accessible from the walkup towers (K-34 in Manaus and a walkup tower 1 km from the K-67 triangle tower in Santarém). In Manaus, diurnal patterns of g_s_ were measured from individual leaves at the top the main crown near the towers from 6:00 to 18:00 using a Li-Cor 6400 XT portable photosynthesis system (Li-Cor, Lincoln^®^, NE, United States). g_s_ measurements on individual leaves were made for 10 min using Li-Cor 6400 XT. The CO_2_ reference concentration was held constant at 400 μmol mol^-1^. T_block_ and photosynthetically active radiation values were set every 15 min to match environmental conditions. Using the Li-Cor 6400 XT we set the T_block_ to achieve a target T_leaf_, based on the infrared radiometers measurements recorded in the CR-3000 datalogger which have a screen that makes possible real time data reads without a computer. In Santarém, g_s_ measurements on individual leaves were made every 2 min using the SD-1 leaf porometer system (Decagon Devices^®^, WA, United States) throughout the day.

### Leaf Water Potential (Ψ_L_) Measurements

In Manaus Ψ_L_ data were collected from three trees together with T_leaf_ measurements (Table 1) to access potential diurnal hysteresis patterns similar to those observed with sap velocity, g_s_, T_air_, T_leaf_, and ΔVPD. Hourly Ψ_L_ measurements (6:00 to 18:00 – 12 h) of healthy leaves without noticeable condensation on the surface of *P. anomala, P. erythrochysa*, and *C. longipendula* were performed in Manaus using a pressure chamber instrument (Model 1000, PMS Instrument Company^®^) connected to a high-pressure nitrogen cylinder. Small branches from the upper tree crowns were removed and a single leaf per tree was used to measure Ψ_L_. The canopy position of each tree was also classified following the crown illumination index proposed by Synnott (1979) (Supplementary Table S1). In this study, the leaf water potential measurements were performed in a single day of September during both 2015 and 2017 dry season.

### Data Analysis

Data were analyzed using IGOR Pro^®^ version 6.3 (WaveMetrics, Inc., United States) and R v. 3.0.2 (R Development Core Team, 2013) software packages. In Manaus, 4-month time series (August to November) were plotted to observe the correlations between T_leaf_ and T_air_ during the 2015 dry season (ENSO). Additionally, the two-dimensional kernel density function (*kde2d*) was used to observe potential offsets between T_leaf_ and T_air_ during the 2015 dry season. In Manaus, the 2015 dry season (ENSO period) was compared with the 2017 dry season (“control scenario”) using hysteresis indexes (H_index_) of the normalized values of T_leaf_, T_air_, and Ψ_L_ for the species *P. anomala, C. longipendula*, and *P. erythrochrysa*. For the normalization of each variable the min-max feature scaling method was used to standardize the range of the raw data. Hysteresis indices were calculated using the shoelace formula (Eq. 5; Braden, 1986), and a paired *t*-test was performed (α = 0.05) to intercompare the H_index_ of the species between the 2015 and 2017 dry seasons. The H_index_ is a measure of the size of the hysteresis loop and enables quantitative comparisons of hysteresis behaviors during, for example, two contrasting periods like El Niño and regular season.

(5) $$A=\frac{1}{2}\left|\sum_{i=1}^{n}x_{i}y_{\mathrm{i+1}}+x_{n}y_{1}-\sum_{i=1}^{n}x_{i}y_{\mathrm{i+1}}-x_{n}y_{1}\right|$$

where: *A* is the area of the polygon, *n* is the number of sides of the polygon, and (*x_i_, y_i_*), *i* = 1, 2,…, *n* are the vertices (or “corners”) of the polygon.

Additionally, normalized sap velocity and T_leaf_ hysteresis parameters of the species *E. cyathiformis, P. anomala*, and *P. erythrochysa* were compared separating the morning and afternoon/night periods during both 2015 and 2017 dry seasons (ENSO and regular season). In the morning period sigmoidal curves were fitted using 15 min interval data of the variables V_s_ and T_leaf_. The statistical parameters of the sigmoidal curves were used to compare the ENSO and the regular season between species. The same approach was done to compare the afternoon/night period of the variables V_s_ and T_leaf_ but using power curves instead of sigmoidal functions.

## Results

### V_s_, T_leaf_, and ΔVPD

**FIGURE 1:**
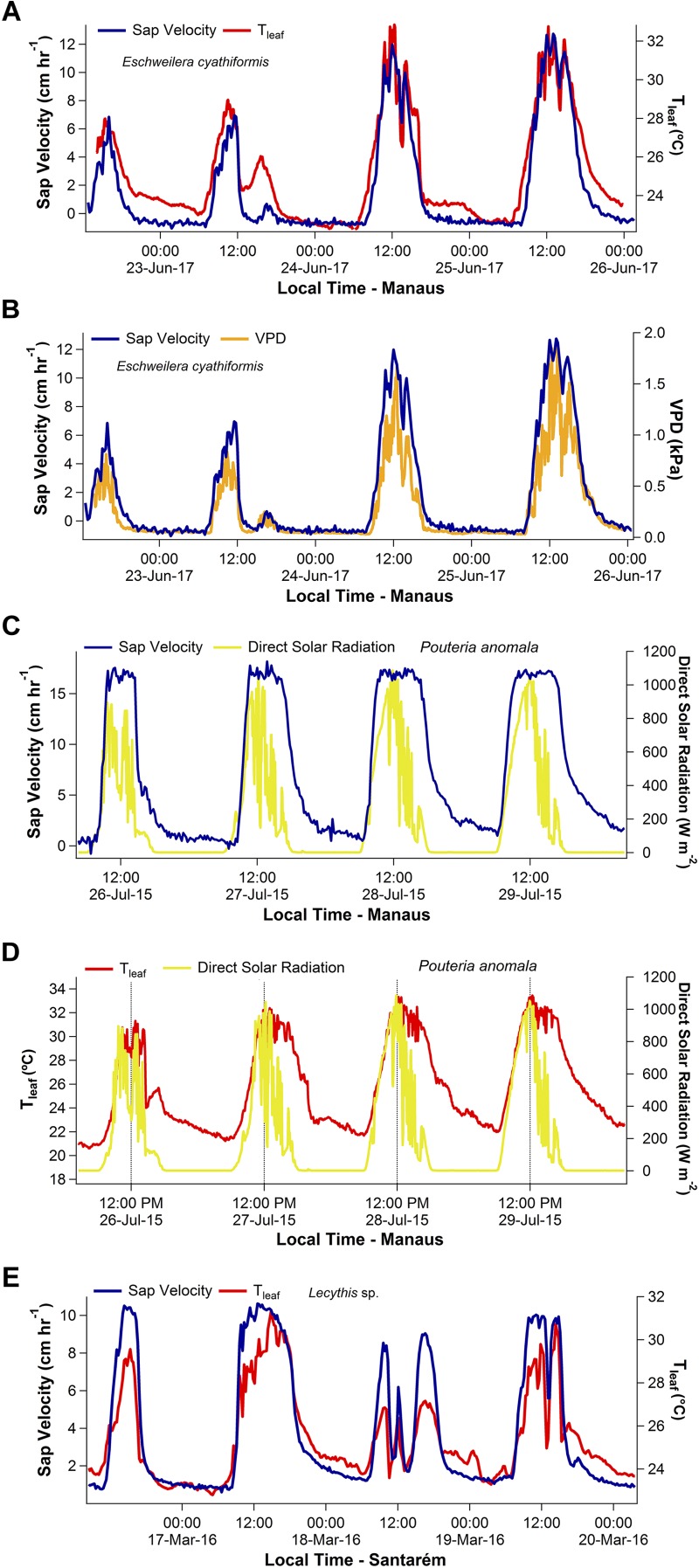
Four-day time series showing the daily patterns of sap velocity (V_s_), leaf temperature (T_leaf_), and vapor pressure deficit (ΔVPD) for the species *Eschweilera cyathiformis, Pouteria anomala*, and *Lecythis* sp. Example of temporal similarities between V_s_ and T_leaf_ are shown in **(A,E)**. Temporal similarities were also observed for V_s_ and ΔVPD **(B)**. A temporal decoupling during the afternoon period was observed between V_s_ and direct solar radiation **(C)**. The contrasting patterns of direct solar radiation and T_leaf_ are also shown in **(D)**.

Representative four-day time series of V_s_ as function of T_leaf_ and ΔVPD for *E. cyathiformis* in Manaus and V_s_ as function of T_leaf_ for *Lecythis* sp. in Santarém are presented in Figure 1. Despite expectations of a significant delay due to the large vertical distance between the observations of V_s_ and T_leaf_, the two variables tightly track each other, during the day and night (Figure 1A,E). Additionally, normalized time series of V_s_ and T_leaf_ of six trees during a two-month period also show this tightly temporal track (Supplementary Figure S4). Moreover, temporal similarities were also graphically observed for the variables V_s_ and ΔVPD (Figure 1B).

Diurnal patterns of direct solar radiation differed from those of T_leaf_ in Manaus, especially during the afternoon period (Figure 1D). Direct solar radiation peaked about mid-day, then declined during the afternoon. In contrast, T_leaf_ and ΔVPD patterns peaked later, during the early afternoon and maintained high values until late afternoon even as solar radiation declined (Figure 1A–E). Thus, for Manaus, on average, a lag of 2 h and 22 min delay occurred between the peaks of direct solar radiation and T_leaf_.

### T_leaf_ and T_air_ Relations

**FIGURE 2:**
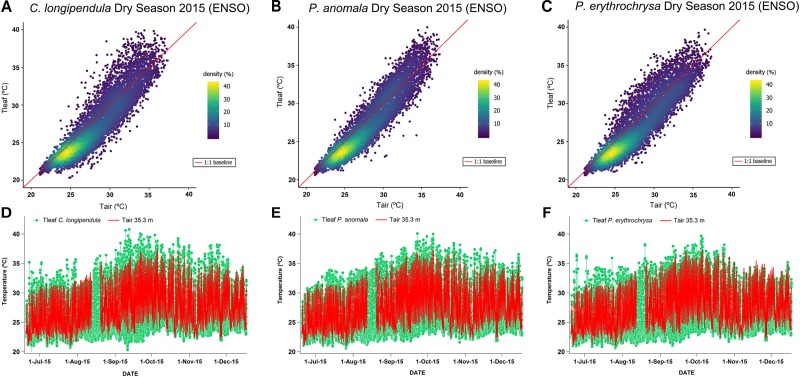
Relationship between T_leaf_ and T_air_ for the species *C. longipendula, P. anomala*, and *P. erythrochrysa* during the 2015 ENSO dry season (August to November) near K-34 tower (Manaus). The scatter plot using the kernel density method allowed to visualize that the observations between T_leaf_ and T_air_ overlaps at maximum rates around 25°C of temperature (40% of density), with the majority of T_leaf_ values lower than T_air_ **(A–C)**. During the months of September and October of 2015, T_leaf_ reached, in some days, more than 40°C for all studied species **(D–F)**. In general, during the 2015 ENSO, maximum values of T_leaf_ for all species were higher than maximum values of T_air_. However, during some days at the end of the 2015 dry season, T_air_ was higher than T_leaf_ for the species *C. longipendula* and *P. erythrochrysa* **(D,F)**.

**FIGURE 3:**
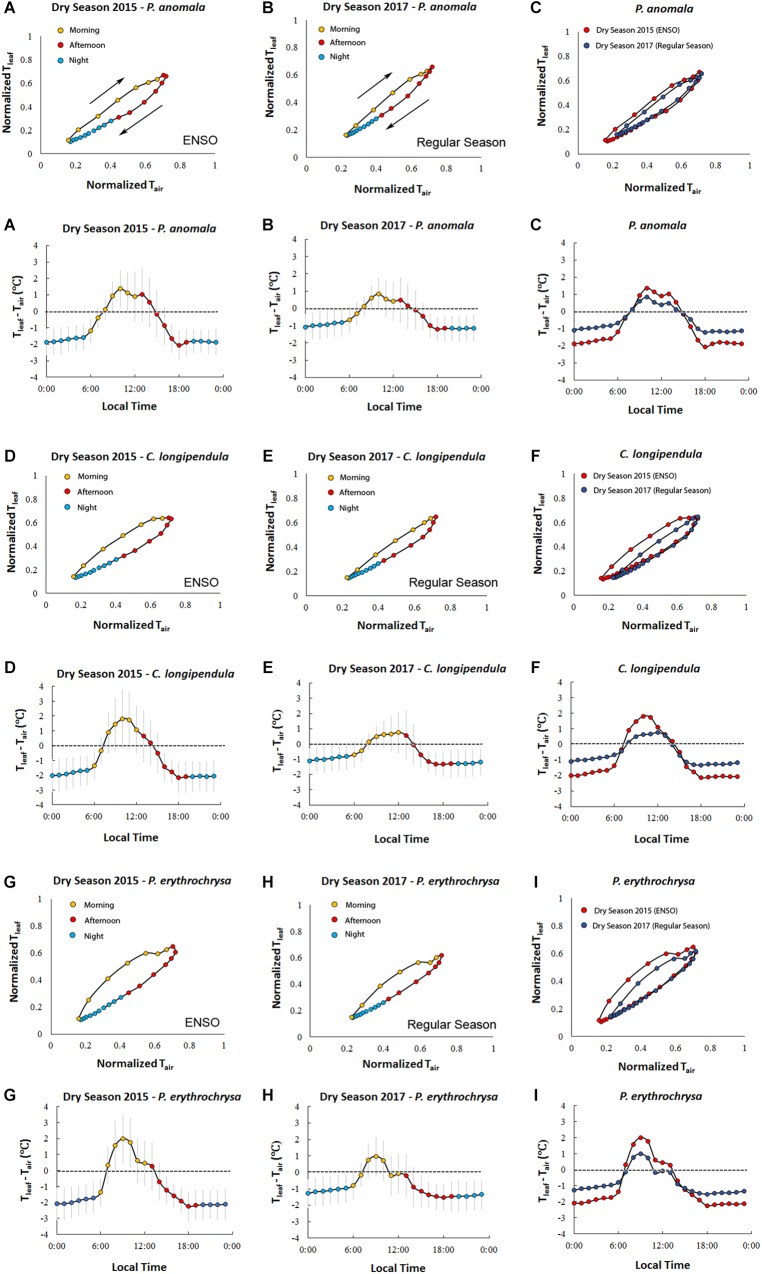
Hourly averages of T_leaf_ and T_air_ for the species *C. longipendula, P. anomala*, and *P. erythrochrysa* in Manaus site during the 2015 dry season (ENSO) and 2017 dry season (“control scenario”). The clockwise hysteresis pattern between T_leaf_ and T_air_ was observed for all the studied trees in Manaus. The orange dots represent the morning period (6:00–12:00), the red dots represent the afternoon period (13:00–19:00), and the blue dots represent the night period (20:00–5:00) **(A,B,D,E,G,H)**. A significant difference (*p* < 0.01) of T_leaf_ and T_air_ hysteresis loops (H_index_) was observed when the 2015 dry season (ENSO period) was compared with the 2017 dry season (“control scenario”) **(C,F,I)**. On average, in both 2015 and 2017 dry season, T_leaf_ was higher than T_air_ during the middle morning to early afternoon, and T_air_ was higher than T_leaf_ in the middle afternoon, night and early morning. The exception was for the species *P. erythrochrysa* where, on average, during the 2017 dry season T_leaf_ was higher than T_air_ only during the morning period (08:00–10:00) **(H)**.

The highest T_leaf_ values during the ENSO in the year of 2015 (2015 dry season – August to November) were observed during the months of September and October (Figure 2). On some days during the 2015 dry season, the differences between T_leaf_ and T_air_ were close to 8°C for some species (average difference between T_leaf_ and T_air_ for all species: 1.65 ± 1.07°C). For the species *C. longipendula* (Figure 2A) the maximum observed T_leaf_ value was 40.78°C (September 12, 2015 – 11:30 local time) and the difference between T_leaf_ and T_air_ was on average 1.70 ± 1.20°C (maximum observed difference 7.43°C); for the species *P. anomala* (Figure 2B) the maximum observed T_leaf_ value was 40.09°C (September 22, 2015 – 15:30 local time) and the difference between T_leaf_ and T_air_ was in average 1.49 ± 0.92°C (maximum observed difference 7.26°C); for the species *P. erythrochrysa* (Figure 2C) the maximum observed T_leaf_ value was 39.67°C (October 4, 2015 – 13:30 local time) and the difference between T_leaf_ and T_air_ was in average 1.75 ± 1.06°C (maximum observed difference 6.39°C). At the end of the 2015 dry season (November), there were some days when T_air_ reached higher values compared to T_leaf_ for the species *C. longipendula* and *P. erythrochrysa* (Figure 2D,F). The 1:1 baseline presented in Figure 2A–C provides a reference to examine deviations of T_leaf_ from T_air_, where the densest observations are between 23 and 26°C with the majority of T_leaf_ values lower than T_air_. This pattern is observed during the night period, when the lowest T_air_ and T_leaf_ values are recorded. In addition, hysteresis patterns between T_leaf_ and T_air_ were observed for the species *P. anomala, C. longipendula*, and *P. erythrochrysa* in Manaus during the 2015 and 2017 dry season (Figure 3). The area of the hysteresis loops (H_index_) of *P. anomala, C. longipendula* and *P. erythrochrysa* during the 2015 dry season (ENSO) were statically larger (*p* < 0.01, α = 0.05, *paired t-*test) than the 2017 dry season (“control scenario”; Figure 3). The H_index_ of the normalized variables T_leaf_ and T_air_ of the species *P. anomala, C. longipendula* and *P. erythrochrysa* during the 2015 dry season was, respectively: 0.0633; 0.0747; 0.0904. Moreover, the H_index_ calculated for *P. anomala, C. longipendula*, and *P. erythrochrysa* for the 2017 dry season was, respectively: 0.0355; 0.0406; 0.0523.

Using hourly averages for all the analyzed species it was possible to observe that generally T_leaf_ was higher than T_air_ from the middle of the morning period until the early afternoon in both the 2015 and 2017 dry seasons (Figure 3). In contrast, T_air_ was predominantly higher than T_leaf_ in the early morning, middle afternoon, and throughout the night in both the 2015 and 2017 dry seasons.

### V_s_-T_leaf_, V_s_-ΔVPD, and V_s_-Direct Solar Radiation Diurnal Hysteresis

**FIGURE 4:**
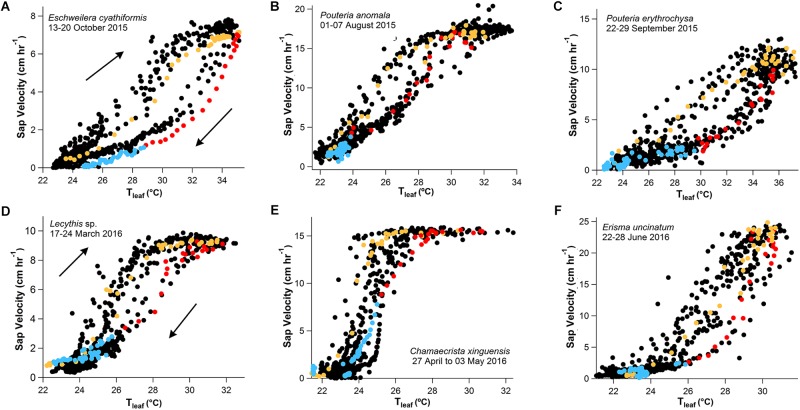
One-week scatter plot data with 15-min observation intervals (black dots) showing the clockwise hysteresis of V_s_ and T_leaf_ for three trees of different species in Manaus **(A–C)**, and Santarém **(D–F)**. The hysteresis phenomenon is represented by a single day of data randomly selected, separated by morning (orange values, 6:00–14:00), afternoon (red values, 14:15–19:00), and nighttime (blue values, 19:15–5:45) periods.

**FIGURE 5:**
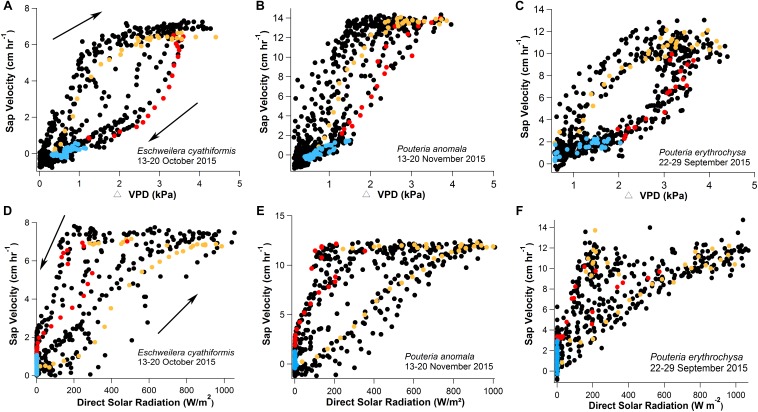
One-week data with 15-min observation intervals showing clockwise hysteresis of V_s_ and ΔVPD **(A–C)** and counterclockwise hysteresis of V_s_ and direct solar radiation **(D–F)** for the species *E. cyathiformis, P. anomala*, and *P. erythrochrysa*. The clockwise and counterclockwise hysteresis phenomena were observed for each tree species using the data of the exact same week (black dots) and day (color dots) separated by morning (orange values, 6:00–14:00), afternoon (red values, 14:15–19:00), and nighttime periods (blue values, 19:15–5:45). During the morning period the V_s_ and ΔVPD clockwise hysteresis pattern showed higher temperature sensitivities compared to afternoon/night periods. In this study, this pattern was called the “g_s_ effect.” During the afternoon period the counterclockwise hysteresis relationship between V_s_ and direct solar radiation showed higher temperature sensitivities compared to morning/night periods and it was called the “VPD effect.” Both observed effects are a result of the Fick’s law of diffusion (transpiration = g_s_ × VPD). For this interpretation, direct solar radiation was considered to have similar temporal patterns with g_s_ by circadian cycles.

As an example, 1 week of V_s_ data were plotted as function of T_leaf_, ΔVPD and direct solar radiation (Figure 4, 5). The clockwise hysteresis in V_s_-T_leaf_ and V_s_-ΔVPD was evident with morning periods showing higher temperature sensitivities than afternoon and night periods and in this study is referred to as the “g_s_ effect” (Figure 4, 5). In Manaus, the scatter plot of V_s_-direct solar radiation revealed a counterclockwise hysteresis pattern, on the same day as the V_s_-ΔVPD clockwise hysteresis (Figure 5). For the same direct solar radiation values, higher V_s_ values in the afternoon were observed relative to the morning period, and in this study this pattern is referred to as the “VPD effect.”

**FIGURE 6:**
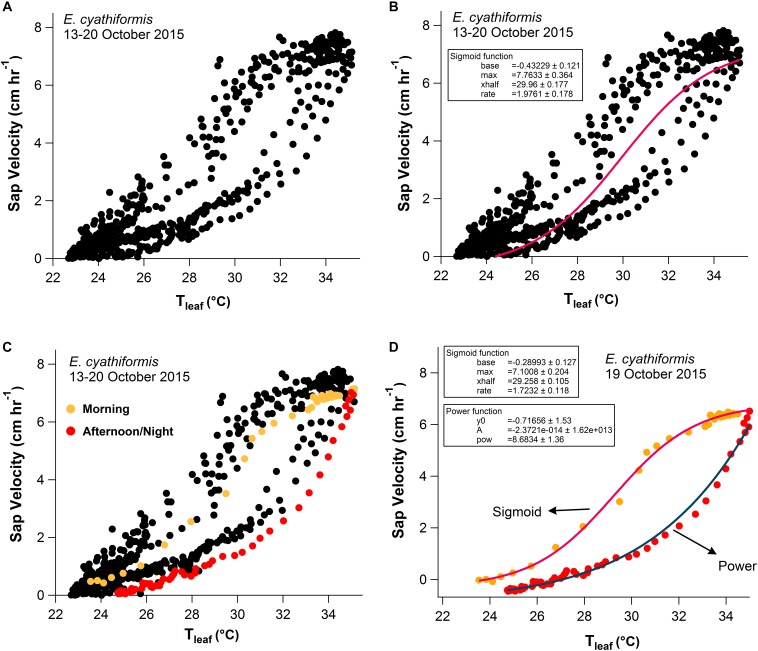
One-week scatter plot data (13–20 October 2015) with 15-min observation intervals for *E. cyathiformis* **(A)**. The sigmoid function presented the best fit for one-week data of sap velocity (V_s_) and T_leaf_ **(A,B)**. A single day data randomly selected, separated by two periods, presented different patterns **(C,D)**. During the morning period (orange dots 6:00–14:00) the sigmoid function presented the best fit *y* = *base* + $\left\{\frac{max}{\left(1+exp\left(\frac{xhalf-x}{rate}\right)\right)}\right\}$ and during the afternoon/night period (red dots 14:15–5:45) the power function presented the best fit *y* = *y*_0_ + *Ax*^pow^ **(D)**.

**FIGURE 7:**
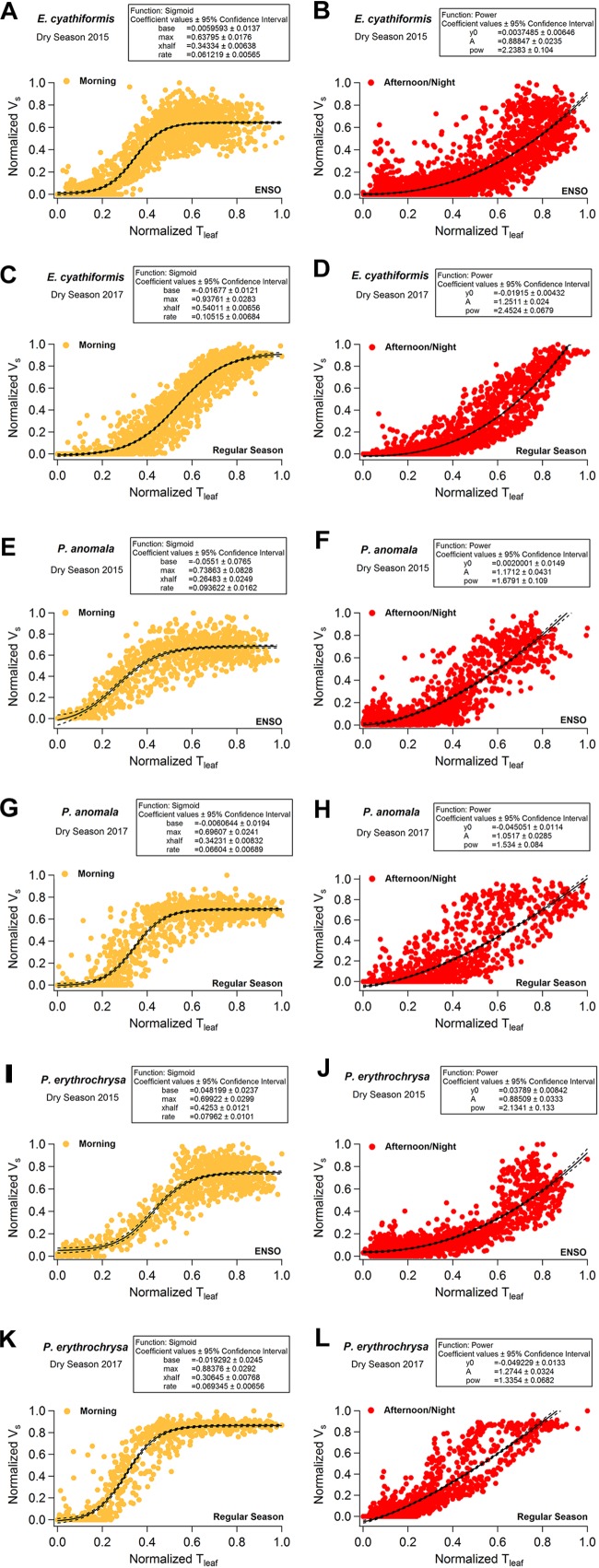
Intercomparison of the hysteretic behavior of the normalized variables V_s_ and T_leaf_ for the species *E. cyathiformis, P. anomala*, and *P. erythrochrysa* during 2015 (ENSO) and 2017 (regular) dry seasons. During the morning period the curve’s maximum values (*max*) of the sigmoid function for variables V_s_ and T_leaf_ were lower during the 2015 dry season (ENSO) in comparison to the 2017 dry season (Regular) for the species *E. cyathiformis* and *P. erythrochrysa* and statically equal for the species *P. anomala* **(A,C,E,G,I,K)**. The inflection point (*xhalf*) of the sigmoidal curves also revealed different patterns for the species when the 2015 dry season was compared to the 2017 dry season. The *xhalf* values were lower for the species *E. cyathiformis* and *P. anomala* during the 2015 ENSO in comparison to the 2017 regular dry season **(A,C,E,G)**. In contrast the *xhalf* value for the species *P. erythrochrysa* was higher during the ENSO in comparison to the 2017 regular dry season **(I,K)**. The statistical values of the power function during the afternoon/night period were relative similar in both ENSO and regular dry season for the species *E. cyathiformis* and *P. anomala* **(B,D,F,H)**. The exception was the species *P. erythrochrysa* which the exponent parameter (*pow*) where higher during ENSO compared to the 2017 regular dry season **(J,L)**.

V_s_ showed a sigmoid dependence on T_leaf_ and ΔVPD including a rapid increase, an inflection point, and a plateau. When V_s_ reached the maximum values for each species, it was insensitive to further increases in T_leaf_ and ΔVPD (Figure 6 and Supplementary Figure S5). The sigmoid pattern was observed in all studied trees (Supplementary Figure S5), although the maximum V_s_ differed between species from 8 to 24 cm h^-1^; detailed daily patterns of V_s_-T_leaf_ revealed a sigmoid increase in V_s_ during the morning period followed by an exponential decrease in the afternoon and throughout the night (Figure 6). During the morning period the curve’s maximum values (*max*) of the sigmoid function for the variables T_leaf_ and V_s_ were lower when the 2015 dry season (ENSO) was compared to the 2017 dry season (regular) for the species *E. cyathiformis* and *P. erythrochrysa* (Figure 7) (curve’s maximum values (*max*): *E. cyathiformis* ENSO (2015): 0.6379 ± 0.017; *E. cyathiformis* regular dry season (2017): 0.9376 ± 0.028; *P. erythrochrysa* ENSO (2015): 0.6992 ± 0.029; *P. erythrochrysa* regular dry season (2017): 0.8837 ± 0.029). For the species *P. anomala* the *max* was statistically equal in both periods (curve’s maximum values (*max*): *P. anomala* ENSO (2015): 0.7386 ± 0.082; *P. anomala* regular dry season (2017): 0.6960 ± 0.024). The inflection point (*xhalf*) of the sigmoidal curves also revealed different patterns between species. The *xhalf* values were lower for the species *E. cyathiformis* and *P. anomala* during the 2015 ENSO in comparison to the 2017 regular dry season (inflection point (*xhalf*): *E. cyathiformis* ENSO (2015): 0.3433 ± 0.006; *E. cyathiformis* regular dry season (2017): 0.5401 ± 0.006; *P. anomala* ENSO (2015): 0.2648 ± 0.024; *P. anomala* regular dry season (2017): 0.3423 ± 0.008. In contrast the *xhalf* value for the species *P. erythrochrysa* was higher during the ENSO in comparison to the 2017 regular dry season (inflection point (*xhalf*): *P. erythrochrysa* ENSO (2015): 0.4253 ± 0.012; *P. erythrochrysa* regular dry season (2017): 0.3064 ± 0.007). The statistical values of the power function during the afternoon/night period was relatively similar in both ENSO and regular dry season for the species *E. cyathiformis* and *P. anomala* (Figure 7). The exception was the species *P. erythrochrysa* which the exponent parameter (*pow*) were higher during ENSO compared to the 2017 regular dry season.

### g_s_-T_leaf_ and Ψ_L_-T_leaf_ Diurnal Hysteresis

Clockwise hysteresis patterns were observed for g_s_-T_leaf_ in Manaus and Santarém (Supplementary Figure S6). For the same T_leaf_, the observed g_s_ values of *E. cyathiformis* and *Manilkara* sp. were greater during the morning period than the afternoon. The maximum observed g_s_ values occurred at a T_leaf_ of 33.3°C in Manaus and 32.6°C in Santarém (Supplementary Figure S6).

**FIGURE 8:**
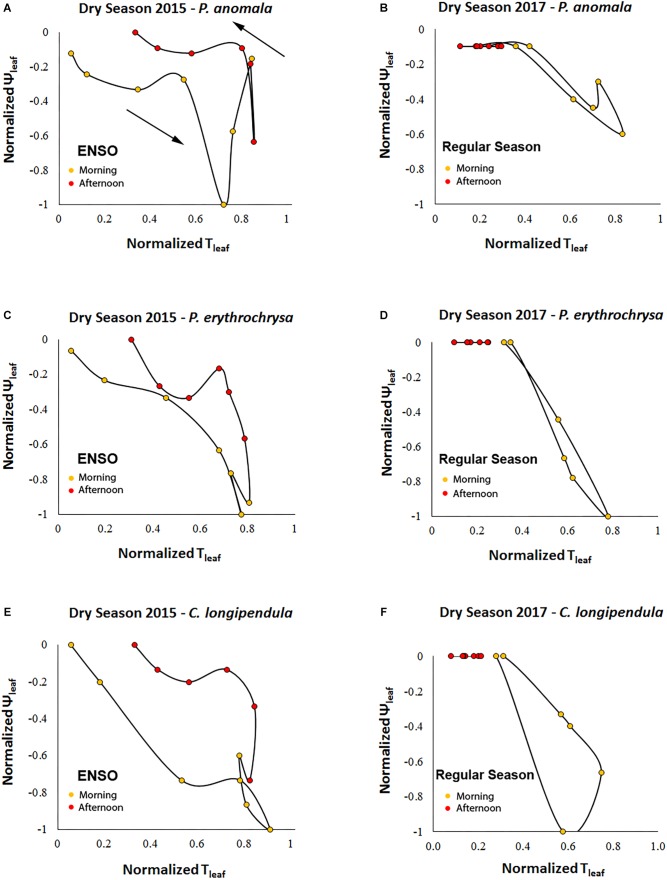
Counterclockwise hysteresis of Ψ_L_ and T_leaf_ for the species *C. longipendula, P. anomala*, and *P. erythrochrysa* in Manaus during the 2015 and 2017 dry seasons. The stomata resistance acts to minimize the water loss during the afternoon periods given the observed counterclockwise pattern for Ψ_L_–T_leaf_. During September 2015 (peak of the El Niño) the H_index_ of the variables Ψ_L_ and T_leaf_ was significant higher **(A,C,E)** compared to September 2017 (regular dry season) (*p* < 0.01, α = 0.05) **(B,D,F)**.

A counterclockwise hysteresis pattern was observed between Ψ_L_ and T_leaf_ (Figure 8). At the same T_leaf_ values, Ψ_L_ were more negative in the morning compared to the afternoon period. During September 2015 (peak of the El Niño) the H_index_ of the variables Ψ_L_ and T_leaf_ were significantly higher compared to September 2017 (regular dry season) (*p* < 0.01, α = 0.05). The H_index_ calculated for the species *P. anomala, C. longipendula*, and *P. erythrochrysa* during the 2015 dry season was, respectively: 0.2379; 0.3116; 0.1605. Moreover, the H_index_ calculated for *P. anomala, C. longipendula*, and *P. erythrochrysa* for the 2017 dry season was, respectively: 0.0642; 0.1596; 0.0324.

## Discussion

### Correlations Between Sap Velocity, T_leaf_, T_air_ and ΔVPD

With a fine-scale measurement resolution of 15 min, temporal similarities between V_s_ and T_leaf_ were observed for all studied species. Delays between V_s_-T_leaf_ and V_s_-ΔVPD, are expected due to the large differences in the heights of the measurements (basal sap velocity versus T_leaf_ in the upper canopy). This type of delays are related to the capacitance of the stems, where the evaporative demand in the branches near the crown are greater than the basal portions of the trunk, with an observed lag in the morning period between these two portions of the tree (Meinzer et al., 2003). Future research should aim to quantify these delays, using for example, simultaneous measurements of V_s_ at DBH height and V_s_ in the branches near the crown, as in Goldstein et al. (1998). At a small time resolution, similar results were previously observed using Granier sap flow system and micrometeorological sensors in French Guiana (Granier et al., 1996), and North Carolina, United States (Oren et al., 1999a). At both sites (F. Guiana and N. Carolina), similar temporal correlations were observed between sap flow and VPD using a measurement resolution of 30 min. A recent study of Bretfeld et al. (2018) showed that V_s_ is largely in phase with VPD_air_ in an 80 year-old-forest which is similar to the observed results of this study. In addition, sigmoid patterns between V_s_-VPD and V_s_-T_leaf_ were also observed for all studied trees (Supplementary Figure S5), as also observed by O’Brien et al. (2004) in tropical species of Costa Rica, and da Costa et al. (2018) in the eastern Amazon. In this study, sap velocity displayed species-specific diurnal hysteresis patterns with a sigmoidal increase during the morning period and an exponential decrease during the afternoon/night period, with statistical differences between the extreme 2015 dry season (ENSO) and a normal 2017 dry season (“control scenario”) (Figure 6, 7).

Given the large diurnal T_leaf_ variation and the exponential dependence of VPD on T_leaf_ (Eqs 2, 3), changes in VPD during the daytime are largely driven by changes in T_leaf_ (Jackson et al., 1981; Ewers and Oren, 2000). The relationship between T_leaf_ and T_air_ represented by T_leaf_-T_air_ offset is a good indicator of water stress in plants (Jackson et al., 1981). Over short time scales (e.g., 10 s) T_leaf_ is also partially related to the leaf mass per area (Michaletz et al., 2016), which can provide new insights about the water dynamics like capacitance. Low temperature values during rainstorms are associated with low VPD and high R_H_ in the Amazon forest (Moradi et al., 2016). Periods of days with the lowest observed V_s_ are related to the lowest observed values of T_leaf_ and ΔVPD. This observation is consistent with previous studies in the Amazon where rain, cloud coverage, and reduced direct solar radiation were found to be the major factors that reduced xylem sap flow rates (Kunert et al., 2017). In addition, other researchers in tropical sites like Costa Rica found that significant leaf wetness also reduces sap flow by up to 28% by impacting VPD (Aparecido et al., 2016).

The positive nighttime V_s_ observed in Manaus and Santarém also apparently followed T_leaf_, similar to that observed by Burgess and Dawson (2004) which found strong positive similarities between nighttime sap flow and VPD in *Sequoia sempervirens*. These observations of positive nighttime V_s_ in the first hours of the night are probably related to the capacitance of stem tissues and radial water transport (Steppe et al., 2012), as well as incomplete stomatal closure (Snyder et al., 2003; Barbour and Buckley, 2007; Dawson et al., 2007) which also allows trees to increase their water content through foliar water uptake (Eller et al., 2013). Another explanation for nighttime transpiration is the water outlet through lenticels, small pores on stem surfaces of many tropical tree species (Roth, 1981). However, it should be noted that no treatment was done to correct V_s_ for small potential offsets (less than 1 cm h^-1^ – lower scales) to estimate the standard deviation of V_s,_ and the possibility of positive nighttime water flow where, in this study, the V_s_ values are generally close to zero.

### Hysteresis Patterns

In the current study, clockwise hysteresis patterns were observed for V_s_-T_leaf_ and V_s_-ΔVPD (Figure 4, 5). Similar results were previously described for sap flow and VPD in tropical forests of Costa Rica (O’Brien et al., 2004) and in tropical secondary forests in Panama (Bretfeld et al., 2018), in temperate forests of Australia (Zeppel et al., 2004), in eastern Amazon trees (Brum et al., 2018) and in a grass-land ecosystem (Zhang et al., 2014). The observed hysteresis phenomenon for sap velocity has been described as a result of the temporal offset of g_s_ that tends to peak, in the tropics, during late morning to mid-day (10:30–12:00) (Slot and Winter, 2017a) (Supplementary Figure S6) and VPD that tends to peak in the early afternoon (13:00–14:30). The hysteresis phenomenon can also be visualized with variables T_leaf_ and direct solar radiation (irradiance). In normal conditions g_s_ respond positively to irradiance (Hennessey and Field, 1991; Gorton et al., 1993; Motzer et al., 2005) and the temporal similarities between these two variables are supposed to be associated with circadian cycles (daily patterns), as demonstrated in some hysteretic behaviors of this study separating the morning, afternoon, and night periods.

In terms of temperature, g_s_ was found to reach a maximum at a T_leaf_ of 31–33°C, which relates with the optimum temperature for photosynthesis (T_opt_) previously determined for many tropical species (Slot and Winter, 2017b). Interestingly, this T_opt_ range seems to match with the inflection point (*xhalf*) in the V_s_-T_leaf_ hysteresis plots in Manaus (Figure 4–7). Thus, we speculate that it may be possible to determine T_opt_ using V_s_-T_leaf_ diurnal hysteresis plots for individual trees. The observed inflection points patterns of V_s_-T_leaf_ during the morning period for the species *E. cyathiformis, P. anomala*, and *P. erythrochrysa* (Figure 7) seems to be influenced by the canopy position and the resistance to drought once, in this study, species-specific shifts emerged when the 2015 ENSO was compared to the 2017 dry season. Additionally, the calculation of an index to quantify the hysteresis loops (H_index_) was performed by this study, similar to a previous approach of Zuecco et al. (2016) which described hysteresis patterns of hydrological variables and runoff events. A quantitative comparison of hysteresis loops from a large number of species using an index approach would be a way of constraining ranges of hysteresis effects in models. Satellite observations revealed that September 2015 exhibited the warmest monthly averaged surface air temperature of any other month over the past 13 years in the Central Amazon (Fontes et al., 2018). These extreme temperatures also modified T_leaf_ patterns, as observed by this study (Figure 3). Higher T_leaf_ values were reflected in a significantly higher H_index_ for the variables T_leaf_-T_air_ and Ψ_L_-T_leaf_ when the 2015 dry season (ENSO period) was compared to the 2017 dry season (“control scenario”). Even though H_index_ for V_s_ was not calculated in this study, we found species-specific shifts in diurnal sap velocity dynamics in both 2015 and 2017 dry seasons contradicting the expected higher values of transpiration during 2015 ENSO compared to other periods due to the high evaporative demand. Brum et al. (2018), for example, found significant differences in the H_index_ for transpiration between dry and wet seasons during the 2015–2016 ENSO in the Eastern Amazon. Nevertheless, the effect of g_s_ during drought events needs to be clarified because trees are expected to show a strong stomatal control under drought conditions, which would offset the expected increase in transpiration H_index_. The higher T_leaf_ values compared to T_air_ during the middle morning to early afternoon observed by this study for the species *P. anomala, C. longipendula*, and *P. erythrochrysa* also contradicts with the expected decrease in T_leaf_ due the transpiration effect. One possible explanation for this pattern is the leaf flushing which is, in the Central Amazon, concentrated in the five driest months (Lopes et al., 2016). On this period, it is possible that the leaf photosynthesis apparatus and tissues aren’t fully developed, reflecting directly in the g_s_ and transpiration patterns. Also, the increasing of light availability in the Amazon forest during the dry season can, maybe, exert an overriding effect in T_leaf_ patterns relative to the cooling effect of transpiration.

Changes in g_s_ are associated with changes in Ψ_L_ via their mutual effects on the balance between V_s_ and transpiration rates. Consistent with the clockwise hysteresis between V_s_-T_leaf_ and V_s_-ΔVPD, a counterclockwise hysteresis pattern was observed between Ψ_L_-T_leaf_. At the same T_leaf_, Ψ_L_ were more negative during morning than afternoon suggesting that partial stomatal closure in the afternoon allows the leaf to recover to less negative Ψ_L_ (Figure 8; Jarvis, 1976). The results suggest that during 2015–2016 ENSO the trees of this study located in a plateau area have a strong isohydric behavior, by reducing stomatal conductance in the warm afternoon periods in order to reduce transpiration rates, thereby minimizing the chances for embolism (Sade et al., 2012; Roman et al., 2015). The same process can be observed in the clockwise hysteresis between V_s_-T_leaf_, and V_s_-ΔVPD where the partial stomatal closure in the afternoon period reduce the V_s_ rates compared to the morning (Figure 4, 5). In this study this pattern was called the “g_s_ effect.” It should be emphasized that the daily hysteresis patterns with the morning, afternoon and night periods separated by colors make it possible to observe both the “g_s_ effect” and “VPD effect” (Figure 5).

Interestingly, the counterclockwise hysteresis pattern of V_s_-direct solar radiation is not driven by the partial stomatal closure in the late morning until the afternoon period, but by the high ΔVPD. At the same solar radiation intensity, higher V_s_ occurs during the afternoon relative to the morning and in this study was called the “VPD effect” (Figure 5). This observations suggests that partial stomatal closure in the afternoon can be offset by the effect of high ΔVPD in maintaining elevated transpiration rates under high afternoon temperatures, as previously shown in other ecosystems (O’Brien et al., 2004; Zeppel et al., 2004; Zhang et al., 2014; Novick et al., 2016; Bretfeld et al., 2018; Brum et al., 2018). Also, these results support recent findings that heat waves can be associated with sustained transpirational cooling as a key mechanism of thermotolerance (Drake et al., 2018). However, the mechanisms of transpiration cooling can be exceeded by the heat intensity during El Niño events, which increase tree mortality in the Amazon forest (Aleixo et al., 2019). Finally, the range of H_index_ for the variables T_leaf_ and T_air_ presented for the first time in this study, can be a useful tool to predict future impacts on tree mortality during extreme drought events in comparison to other periods.

The observed differences of the maximum V_s_ rates (curve’s maximum values (*max*) of sigmoidal functions during the morning period – Figure 7)) between species can be related to the diameter of the vessels (Dünisch and Morais, 2002), wood density (Eller et al., 2018) and with the susceptibility to embolism (Lovisolo and Schubert, 1998). Tree height is also an important factor which influences sun exposure and therefore the temperature of the leaves and transpiration rates (Goldstein et al., 1998). In Manaus, *E. cyathiformis* was the thinnest and shortest studied tree with 14.3 cm of DBH and 19.8 m of height and was the tree with the lowest observed V_s_ rates (∼8 cm h^-1^) during ENSO (Figure 4). In contrast, the *Pouteria* genus (*P. anomala* and *P. erythrochysa*) have large DBHs (35.3 cm and 36.5 cm) and high rates of V_s_ during ENSO (18 and 12 cm h^-1^, respectively) (Figure 4). This is consistent with other studies where DBH, height and sap flow showed a positive correlation (Motzer et al., 2005). Likewise, the same correlations were observed in Santarém, where the larger DBHs showed the higher sap velocity. Also, the observed differences in curve’s maximum values (*max*) of sigmoidal curves observed for V_s_ and T_leaf_ during ENSO period and the regular dry season (Figure 7), may be associated with the high range of functional traits and susceptibility to embolism of some trees in the Amazon forest along hydro-topographic gradients (Cosme et al., 2017; Oliveira et al., 2019). Other important issue is that mortality rates during droughts are substantially higher for large DBH’s (Meakem et al., 2017), and maybe this is potentially aggravated by high transpiration rates and the crown exposure to the direct light. Another study of rain exclusion in the eastern Amazon by Rowland et al. (2015) support this observation. However, other factors are also involved since it has been shown that early-successional forests experienced more drought stress than trees in late-successional forests (Bretfeld et al., 2018). In fact, more studies are needed in the tropics, especially in the Amazon, due to the large diversity of terrestrial plants (Ter Steege et al., 2013), the wide range of functional traits and evolutionary strategies to avoid cavitation, carbon starvation, and other aspects related to drought which can modify T_leaf_ and V_s_ patterns.

## Conclusion

For the first time in the Amazon forest, the quantitative differences and the hysteresis pattern between T_leaf_ and T_air_ were demonstrated and compared during the 2015 (ENSO) and 2017 (“control scenario”) dry seasons. The relationship between T_leaf_ and T_air_ was significantly different between these two periods and, in general, T_leaf_ was higher than T_air_ during the middle morning to early afternoon. The use of the variable T_leaf_ together with T_air_ are extremely important to ecophysiological observations due to the differences in terms of magnitude and temporal patterns. Also, T_leaf_ is an important variable to estimate the true water vapor pressure gradient between the substomatal cavity and the boundary layer of the air near the leaf surface (ΔVPD). Moreover, sap velocity displayed species-specific diurnal hysteresis patterns that were strongly linked to g_s_ and VPD and reflected by changes in T_leaf_. In the morning, g_s_ was linearly related to T_leaf_ and sap velocity displayed a sigmoidal relationship with T_leaf_. In the afternoon, stomatal conductance declined as T_leaf_ approached a daily peak, allowing Ψ_L_ to begin recovery, while sap velocity declined with an exponential relationship with T_leaf_. Hysteresis indices (T_leaf_ : T_air_ and T_leaf_ : Ψ_L_) were much more pronounced during the ENSO event than during a typical dry season and varied between species, which reflects species-specific capacitance and tree hydraulic traits. Future research may address a new modeling approach using the magnitude of the hysteresis loops (H_index_) to measure, for example, the intensity of the droughts and how it impacts plant communities. Finally, the hysteretic behavior of the transpiration separated by morning, afternoon and night periods is the key to understand the complexity of this process in a changing climate and improve the current models.

## Author Contributions

BG, KJ, RN-J, IS-F, CF, LC, JH, JC, NH, TD, and NM performed the experiments and analyzed the data. JC, NH, TD, and NM planned and designed the experiments. BG and KJ wrote the manuscript. CF, AA, JW, BN, CV, DC, GS, CK, and NM improved the manuscript.

## Conflict of Interest Statement

The authors declare that the research was conducted in the absence of any commercial or financial relationships that could be construed as a potential conflict of interest.

## Footnotes

1 https://www.apogeeinstruments.com/irr-calculators/
