# Supplementary figures and images for: Species-Specific Shifts in Diurnal Sap Velocity Dynamics and Hysteretic Behavior of Ecophysiological Variables During the 2015–2016 El Niño Event in the Amazon Forest
Source: Front Plant Sci. 2019 Jun 28;10:830. doi: 10.3389/fpls.2019.00830 (PMC6611341; doi:10.3389/fpls.2019.00830)

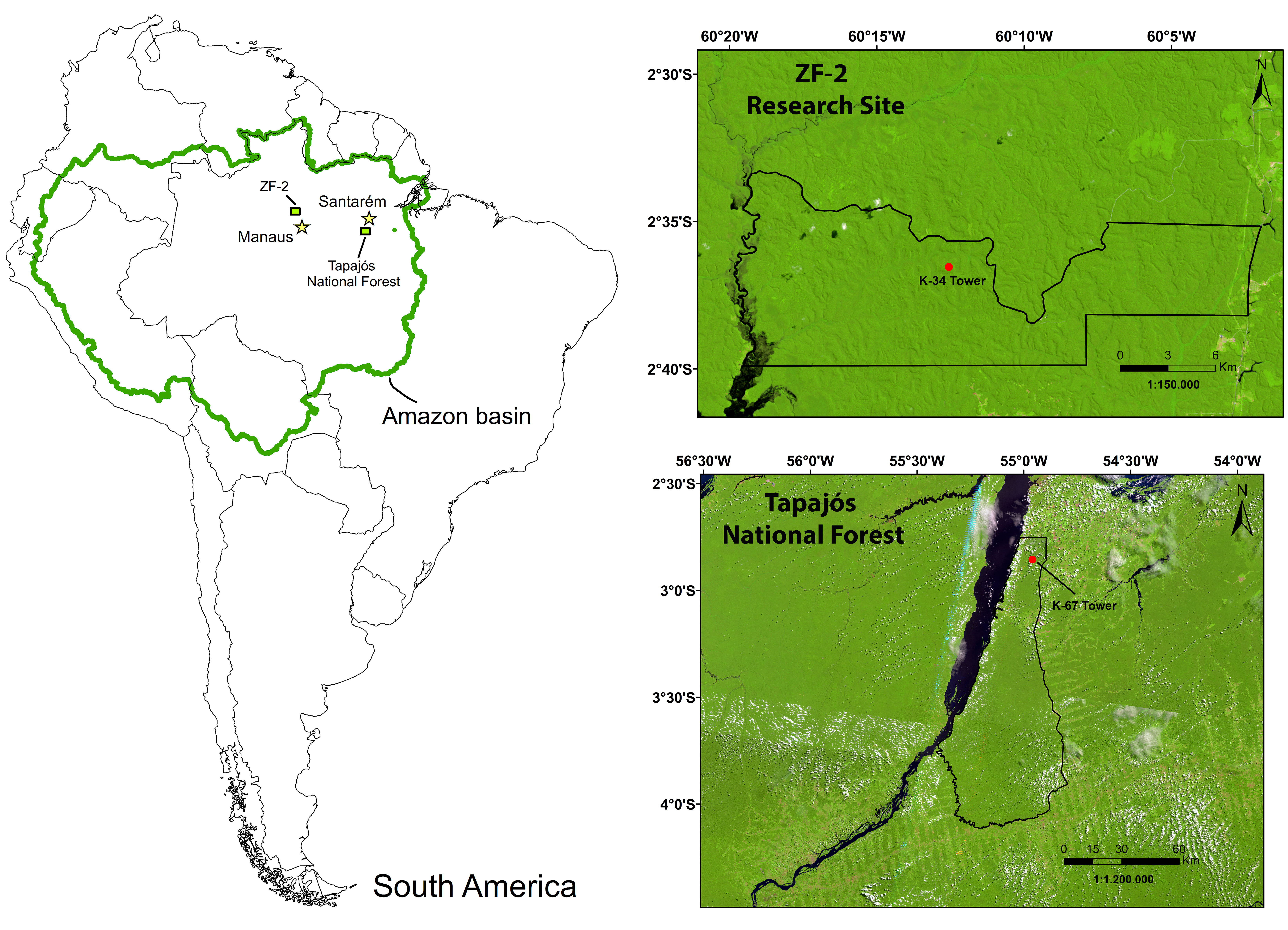

Supplement: Supplementary file 1 [file Image_1.TIF]

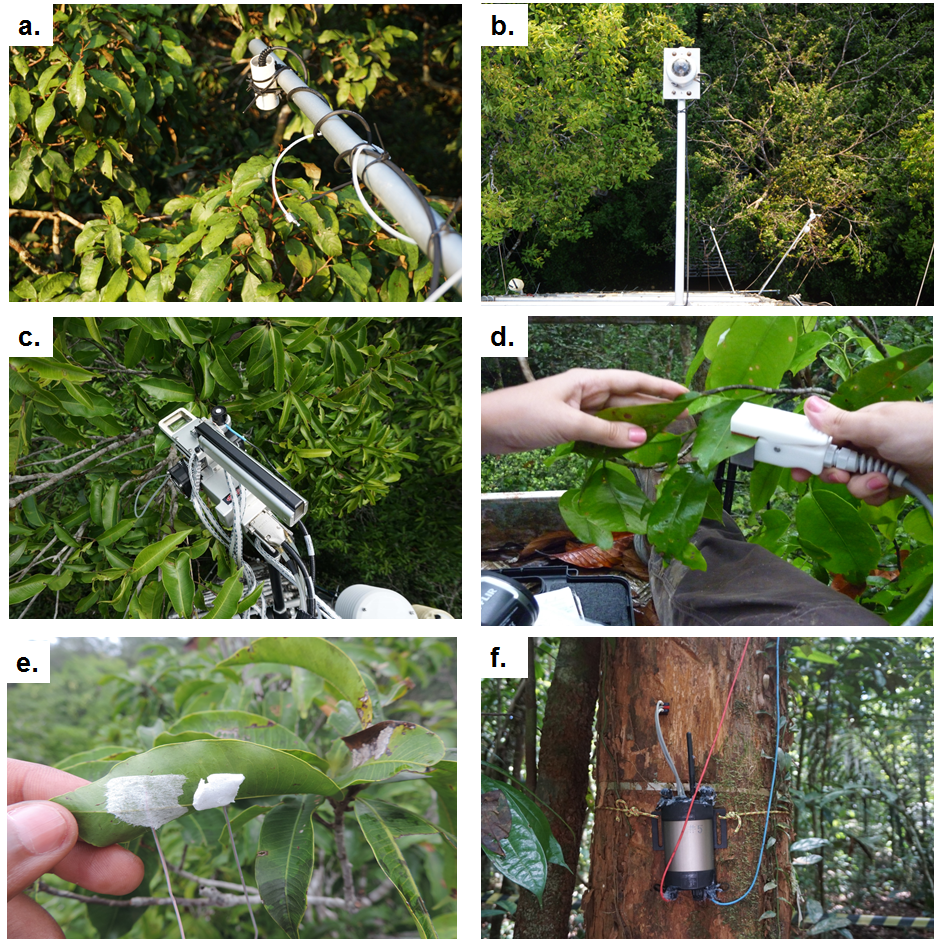

Supplement: Supplementary file 2 [file Image_2.TIF]

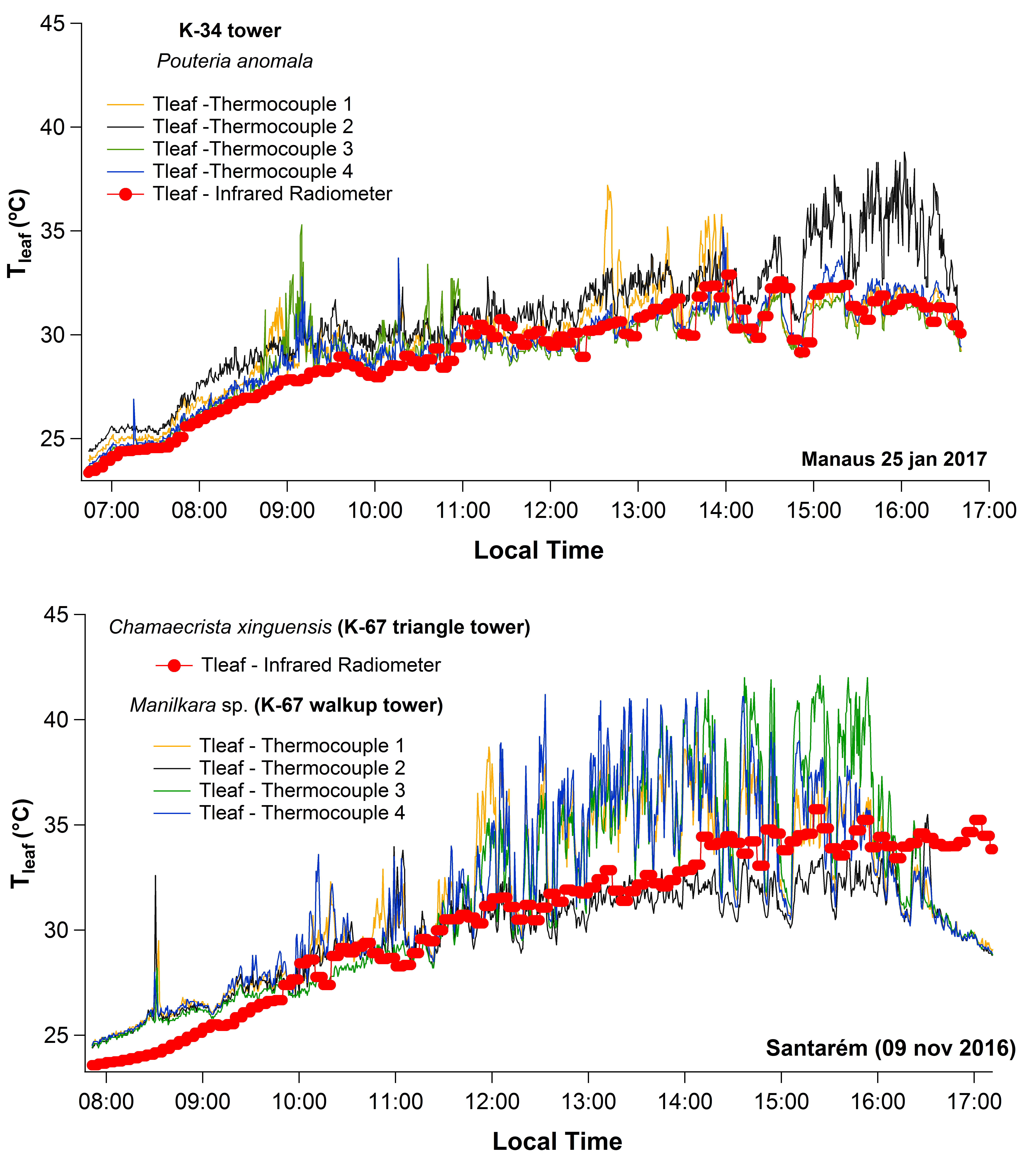

Supplement: Supplementary file 3 [file Image_3.TIF]

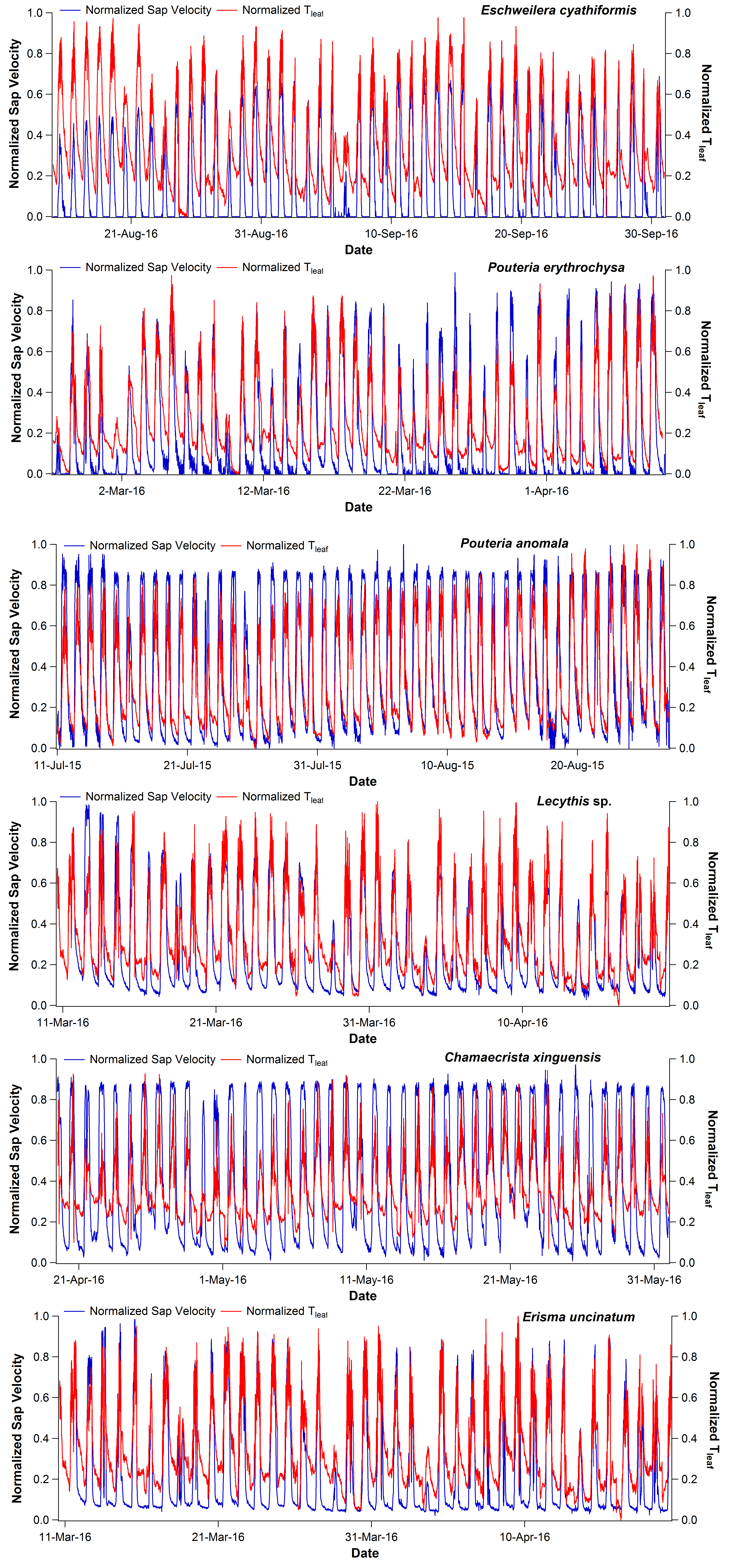

Supplement: Supplementary file 4 [file Image_4.TIF]

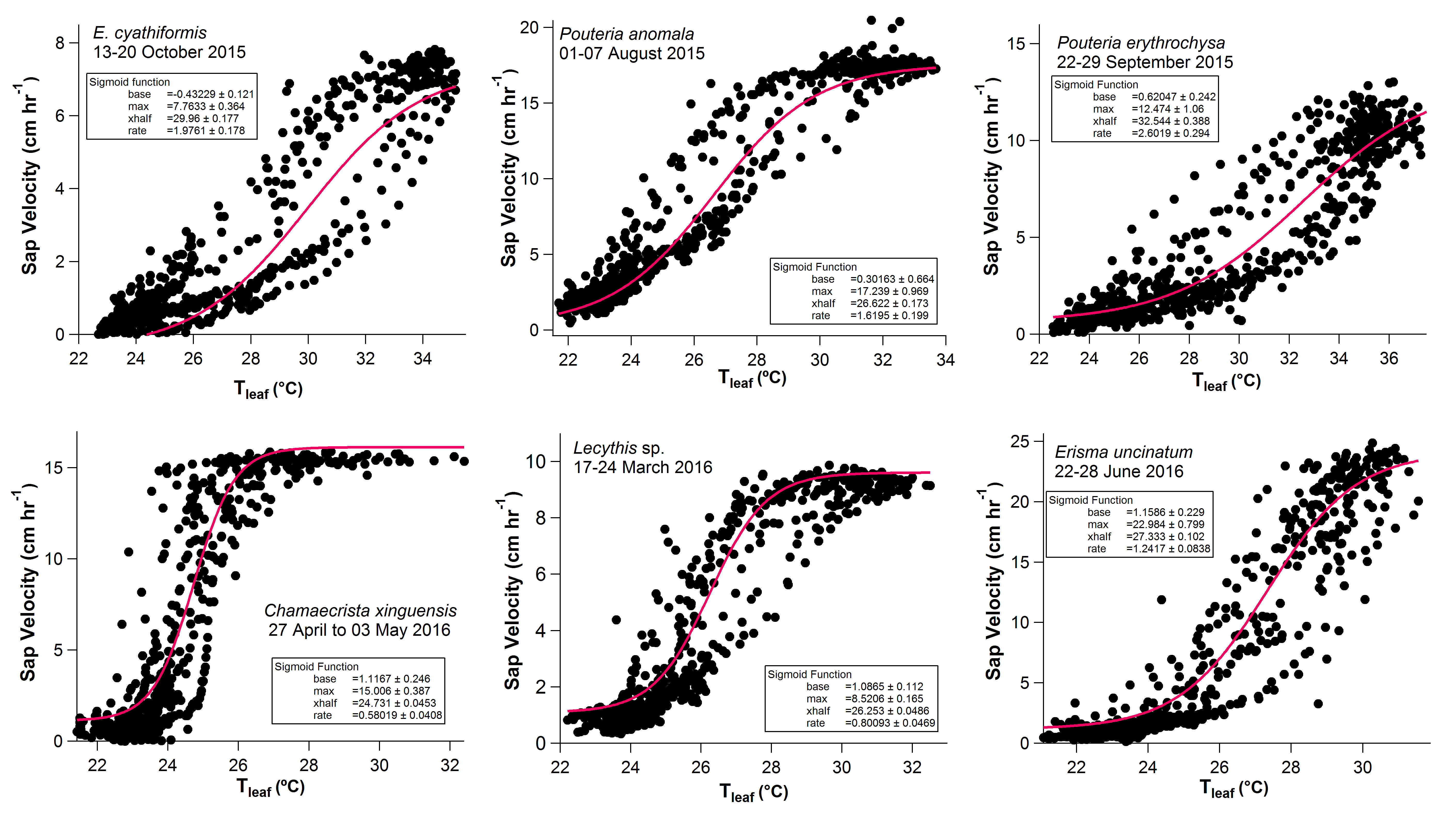

Supplement: Supplementary file 5 [file Image_5.TIF]

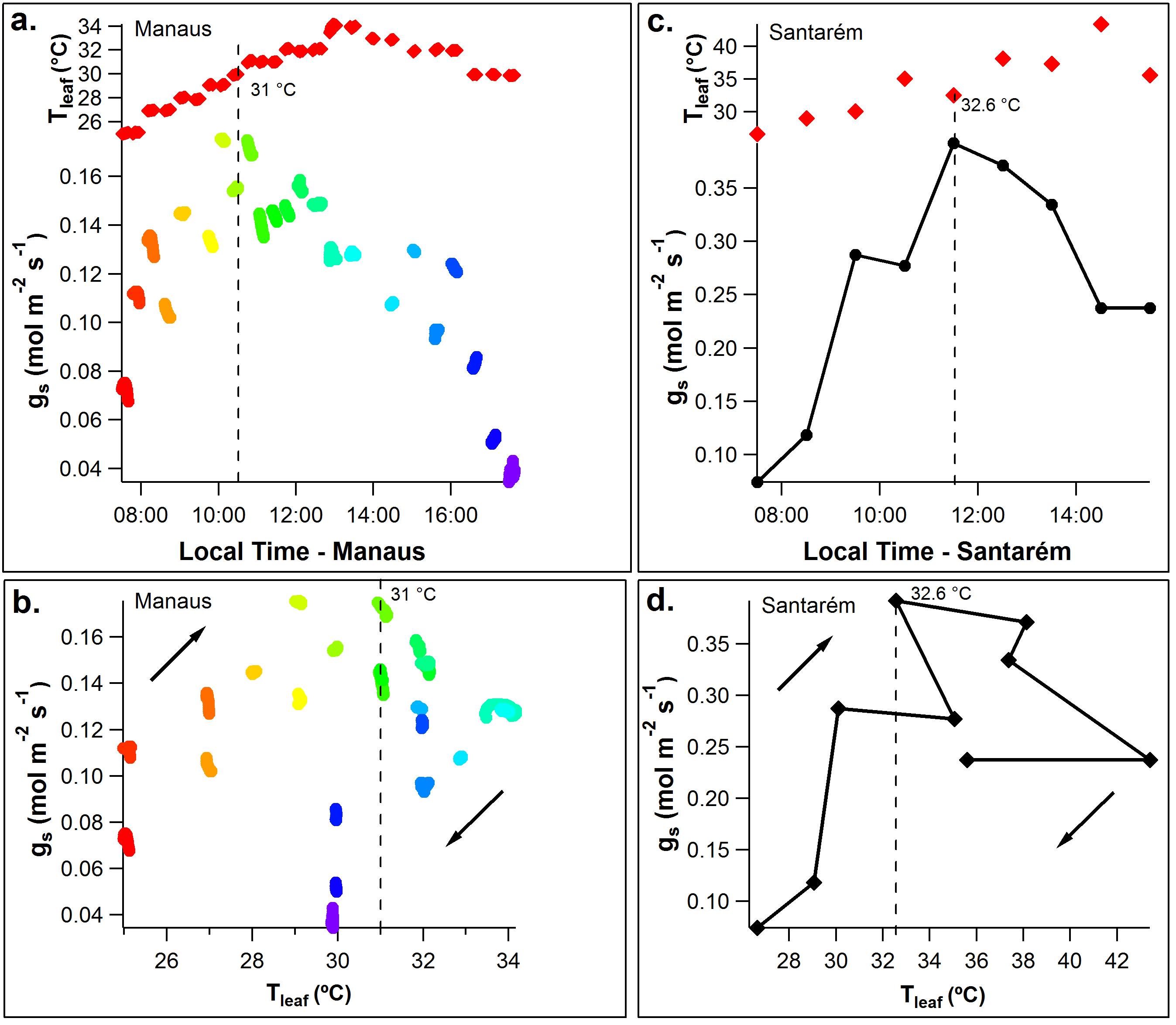

Supplement: Supplementary file 6 [file Image_6.TIF]
