# Supplementary material for: Species-Specific Shifts in Diurnal Sap Velocity Dynamics and Hysteretic Behavior of Ecophysiological Variables During the 2015–2016 El Niño Event in the Amazon Forest
Source: Front Plant Sci. 2019 Jun 28;10:830. doi: 10.3389/fpls.2019.00830 (PMC6611341; doi:10.3389/fpls.2019.00830)
Supplement: Supplementary file 7 [file Table_1.docx]

| **Tree Species** | **Height (m)** | **Crown Ilumination Index (Adapted from Synnott, 1979)** |
| --- | --- | --- |
| *Pouteria anomala* | 31.0 | **4** - Crown completely exposed to vertical light (e.g. canopy tree) |
| *Couepia longipendula* | 23.9 | **2b** - Some lateral light |
| *Pouteria erythrochrysa* | 29.3 | **3b** - High but not full vertical illumination (e.g. lower canopy tree) |

**Supplementary Table S1**. Crown illumination index of three species located in K-34 site (Manaus) where Ψ_L_ experiments were performed during the 2015 and 2017 dry seasons.
