# Supplementary material for: Species-Specific Shifts in Diurnal Sap Velocity Dynamics and Hysteretic Behavior of Ecophysiological Variables During the 2015–2016 El Niño Event in the Amazon Forest
Source: Front Plant Sci. 2019 Jun 28;10:830. doi: 10.3389/fpls.2019.00830 (PMC6611341; doi:10.3389/fpls.2019.00830)
Supplement: Supplementary file 8 [file Data_Sheet_1.docx]

Supplementary Material

**Species-specific shifts in diurnal sap velocity dynamics and hysteretic behavior of ecophysiological variables during the 2015-2016 El Niño event in the Amazon forest**

**Bruno O. Gimenez^1*^**†**, Kolby J. Jardine^2^**†**, Niro Higuchi^1^, Robinson I. Negrón-Juárez^2^, Israel de Jesus Sampaio-Filho^1^, Leticia O. Cobello^1^, Clarissa G. Fontes^3^, Todd E. Dawson^3^, Charuleka Varadharajan^2^, Danielle S. Christianson^2^, Gustavo C. Spanner^1^, Alessandro C. Araújo^4^, Jeffrey M. Warren^5^, Brent D. Newman^6^, Jennifer A. Holm^2^, Charles D. Koven^2^, Nate G. McDowell^7^, and Jeffrey Q. Chambers^2,8^**

^1^National Institute of Amazonian Research (INPA), Manaus, Brazil; ^2^Climate and Ecosystem Sciences Division, Lawrence Berkeley National Laboratory, Berkeley, CA, USA; ^3^Department of Integrative Biology, University of California Berkeley, Berkeley, CA, USA; ^4^Embrapa Amazônia Oriental, Belém, Brazil; ^5^Environmental Sciences Division and Climate Change Science Institute, Oak Ridge National Laboratory, Oak Ridge, TN, USA; ^6^Earth and Environmental Sciences Division, Los Alamos National Laboratory, Los Alamos, NM, USA; ^7^Pacific Northwest National Laboratory, Richland, WA, USA; ^8^Department of Geography, University of California Berkeley, Berkeley, CA, USA

*** Correspondence:** [bruno.oliva.gimenez@gmail.com](mailto:bruno.oliva.gimenez@gmail.com)

† These authors have contributed equally to this work

**Supplementary Figure S1** Map of the study areas developed using Landsat 8 satellite images (available in <https://earthexplorer.usgs.gov/>) where the K-34 tower and K-67 tower are located. The K-34 tower is in the central Amazon, near the city of Manaus, Brazil. The K-67 tower is in the north side of Tapajós National Forest (eastern Amazon) near the city of Santarém, Brazil.

**Supplementary Figure S2** Images of the sensor network used to study the dependencies of tree physiology on environmental conditions in Manaus and Santarém, Brazil. (**a**) T_leaf_ measurements using infrared radiometer sensors, Apogee®; (**b**) direct solar radiation equipment - SPN1 sunshine pyranometer, Delta-T Devices®; (**c**) leaf gas exchange system – LiCor 6400XT, Lincoln®, NE, USA; (**d**) leaf stomatal conductance measurements (porometer) - Decagon Devices®, WA, USA; (**e**) T_leaf_ thermocouples measurements - OM-CP-OCTTEMP-A Nomad®, Omega Engineering; (**f**) sap velocity system - SFM1, ICT international®.

**Supplementary Figure S3** Intercomparisons between T_leaf_ measurements using infrared and leaf thermocouples. In Manaus, the results show good agreement between the two methods for T_leaf_ estimation with values generally falling within the range of the four different leaf thermocouples. In Manaus both methods were tested using the same tree species (*P. anomala*), in which was possible because the proximity of the crown to the K-34 walkup tower. In Santarém, the T_leaf_ observations using the infrared radiometers were underestimated due to the larger distance between the sensors mounted on the K-67 triangle tower and the crowns of the trees. While in Manaus the target areas were up to 2.4 m^2^, which consisted mainly of sun-exposed leaves, the target areas in Santarém were up to 65.4 m^2^ which consisted of both shade and sun-exposed leaves, and branches. An underestimate values of T_leaf_ in Santarém was suggested using leaf thermocouples installed in upper canopy leaves of *Manilkara* sp. (~30 m height) accessible next to a nearby walkup tower. The values of diurnal thermocouple data of *Manilkara* sp. were compared with the infrared radiometers mounted on the K-67 triangle tower with the field of view to the tree *Chamaecrista xinguensis* (~30 m height). On 09 November 2016 maximum afternoon T_leaf_ determined by infrared radiometers on the K-67 triangle tower were 33-35°C for *Chamaecrista xinguensis* whereas maximum afternoon T_leaf_ determined by leaf thermocouples on the nearby walkup tower were 40-42°C for *Manilkara* sp. Thus, the T_leaf_ data in Manaus is considered quantitative whereas the T_leaf_ data in Santarém is considered more qualitative but expected to follow the temporal patterns of the actual T_leaf_.

**Supplementary Figure S4** Normalized long time series (2 months) of V_s_ and T_leaf_ of six trees (*E. cyathiformis*, *P. anomala*, and *P. erythrochysa* in Manaus, and *C. xinguensis*, *Lecythis* sp., and *E. uncinatum* in Santarém), with the peaks and valleys of these two variables coinciding temporally.

**Supplementary Figure S5** One-week scatter plot data with 15-minute observation intervals during the 2015-2016 ENSO for *E. cyathiformis*, *P. anomala*, and *P. erythrochysa* in Manaus, and *C. xinguensis,* *Lecythis* sp. and *E. uncinatum* in Santarém. The sigmoid function presented the best fit for one-week data of sap velocity as function of T_leaf_. Four parameter logistic regressions (4PL) were fitted for each species using the following equation: $y=base+ \left\{ \frac{max}{\left( 1+exp\left( \frac{xhalf-x}{rate} \right) \right)} \right\}$

**Supplementary Figure S6** Diurnal patterns of g_s_ for *E. cyathiformis* in Manaus (**a, c**) and for *Manilkara* sp. in Santarém (**b, d**). The g_s_ peak for *E. cyathiformis* in Manaus was 31 ºC (~ 10:20 Local Time) and the g_s_ peak for *Manilkara* sp. In Santarém was 32.6 ºC (~ 11:30 Local Time), both in the morning period. Additionally, clockwise hysteresis patterns of the variables g_s_ and T_leaf_ was observed in the two sites (**c, d**).
